# Behavior Deterioration and Microstructure Change of Polyvinyl Alcohol Fiber-Reinforced Cementitious Composite (PVA-ECC) after Exposure to Elevated Temperatures

**DOI:** 10.3390/ma13235539

**Published:** 2020-12-04

**Authors:** Qing Wang, Boyu Yao, Runze Lu

**Affiliations:** 1 School of Civil Engineering, Guangzhou University, Guangzhou 510006, China; 2111816243@e.gzhu.edu.cn; 2 International Department, The Affiliated High School of SCNU, Guangzhou 510630, China; lurz.eric2019@gdhfi.com

**Keywords:** engineered cementitious composites, elevated temperature, polyvinyl alcohol fiber, residual mechanical properties, microstructure

## Abstract

In the case of fire, explosive spalling often occurs in cementitious composites due to dense microstructure and high pore-pressure. Polymer fibers were proved to be effective in mitigating such behavior. However, deterioration of these fiber-reinforced cementitious composites inevitably occurs, which is vital for the prediction of structural performance and prevention of catastrophic disaster. This paper concentrates on the behavior and mechanism of the deterioration of polyvinyl alcohol fiber-reinforced engineered cementitious composite (PVA-ECC) after exposure to elevated temperatures. Surface change, cracking, and spalling behavior of the cubic specimens were observed at room temperature, and after exposure to 200 °C, 400 °C, 600 °C, 800 °C, and 1200 °C. Losses in specimen weight and compressive strength were evaluated. Test results indicated that explosive spalling behavior was effectively prevented with 2.0 vol% polyvinyl alcohol fiber although the strength monotonically decreased with heating temperature. X-ray diffraction curves showed that the calcium hydroxide initially decomposed in the range of 400–600 °C, and finished beyond 600 °C, while calcium silicate hydrate began at around 400 °C and completely decomposed at approximately 800 °C. Micrographs implied a reduction in fiber diameter at 200 °C, exhibiting apparent needle-like channels beyond 400 °C. When the temperature was increased to 600 °C and above, the dents were gradually filled with newly produced substance due to the synergistic effect of thermal expansion, volume expansion of chemical reactions, and pore structure coarsening

## 1. Introduction

Concrete, a traditional building material, has been recognized as a quasi-brittle material with low ductility and high brittleness, which is harmful to the serviceability and durability of concrete structures. To overcome these drawbacks, engineered cementitious composites (ECC) with strain-hardening features have been developed over the last three decades [1,2,3]. It was first designed according to micromechanical mechanics [4,5] by adding a small amount of ductile fiber. Polyvinyl alcohol (PVA) fibers are often incorporated thanks to their homogenous distribution and significant bridging effect among cement pastes [6,7]. The ultimate strain of PVA fiber-reinforced ECC (PVA-ECC) under direct tension can reach 3–8% according to previous research [8,9,10], with all crack widths smaller than 100 μm [11]. Such excellent properties enable PVA-ECC to be broadly applied in construction fields, such as dam retrofitting, bridge deck overlaying, pavement linking, coupling beams installation, and masonry structures strengthening [12,13,14,15,16,17,18,19].

However, wider applications would increase the probability of PVA-ECC encountering the risk of fire or high temperature. When on fire, the maximum indoor temperature of a building structure could reach as high as 800 °C to 1000 °C, the mechanical properties of cement-based materials become seriously damaged, and the bearing capacity and deformability of structure elements will also be greatly weakened. During the last decade, over 310,000 fire disasters occurred in China, i.e., nearly 8.5 cases per day, and more than 1269 people died in home fires in the year of 2016, not to mention the economic loss. Therefore, fire resistance and the degradation mechanism of cementitious materials are of vital importance to structural safety and have attracted ever-increasing attention recently [20,21,22,23]. Concrete, especially ultra-high-performance concrete, is often susceptible to explosive spalling under fire conditions, with structural component pieces breaking off or flaking at a high speed. Such phenomena not only lead to a loss in cross-section, but also expose the embedded reinforce bars directly to fire. Therefore, behavior responses of PVA-ECC under high temperatures are of great significance not only for material reliability prediction and direction, but also for structure safety and fire-resistance design.

**Figure 1:**
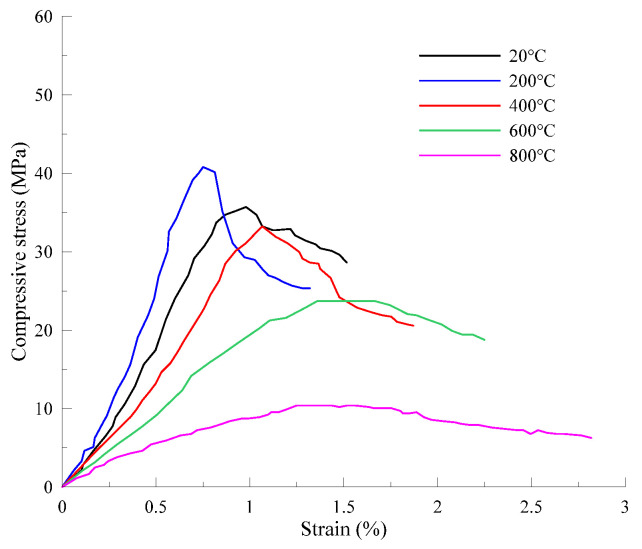
Compressive stress–strain curves of specimens after exposure to high temperatures for different heating durations [25].

For concrete, the residual compressive strength after experiencing 400 °C is generally reported around 80%. However, the reduction rate of strength beyond this temperature increases dramatically, resulting in less than 10% of residual strength compared to that at ambient conditions in the range of 800–900 °C [24]. Figure 1 shows the typical compressive stress–strain curves of PVA-ECC after exposure to various temperatures for 1 h [25].

Nonlinear behavior was observed for all curves and the peak stress and stiffness decreased with the increase in heating temperature (except for 200 °C), whilst the corresponding strain increased. Such phenomena were also reported by other authors [26,27]. It is also noticed that all specimens subjected to 200 °C for different durations exhibited higher strength and stiffness under compression loads compared to the control group [28]. Herein, a longer exposure duration increases the peak stress but decreases the corresponding strain at the same time [25]. Moreover, Şahmaran et al. [29] investigated the effect of fly ash on the residual properties of ECC exposed to high temperatures up to 800 °C and found that micro PVA fiber improved the fire resistance of matrix and eliminated the explosive spalling behaviors. However, the mechanism of fire resistance improvement by PVA fiber has not been fully understood, especially for the enhancement effect at about 200 °C.

The behaviors of PVA-ECC under fire/high temperatures are believed to be related to microstructure changes in cementitious composites at elevated temperatures. For instance, the decompositions of ettringite (3CaO∙Al_2_O_3_∙3CaSO_4_∙32H_2_O), calcium hydroxide (CH), and calcium silicate hydrate (C-S-H) gels were reported to be initiated at 70, 430, and 600 °C, respectively [30]. Such decompositions inevitably lead to chemical composition variation and interfacial transition zone alteration under a microscope. The observation in micrographs of fire-damaged PVA-ECC may help to comprehend the mechanism. In terms of PVA fibers, they melt at 230–250 °C and lose bridging ability between cement pastes. Instead, channels with the shape of PVA fibers are created, which provide extra passages for steam to escape under elevated temperatures.

As aforementioned, the fire safety of PVA-ECC materials in construction should be greatly emphasized. Herein, the connections between behavior deterioration under fire (color change, surface cracking, explosive spalling phenomenon, mechanical properties loss, etc.) and microstructure change need to be established. To achieve this, changes in mechanical performance and microstructure of PVA-ECC subjected to different elevated temperatures were focused on in the present paper. Color changes and surface cracking behaviors of specimens were visually observed. Compressive strengths after exposure to various temperatures were tested. Furthermore, X-ray diffraction (XRD) tests were conducted and micrographs were also taken by means of SEM to illustrate the behaviors in the macroscope.

## 2. Materials and Methods

### 2.1. Material and Mix Proportions

**Figure 2:**
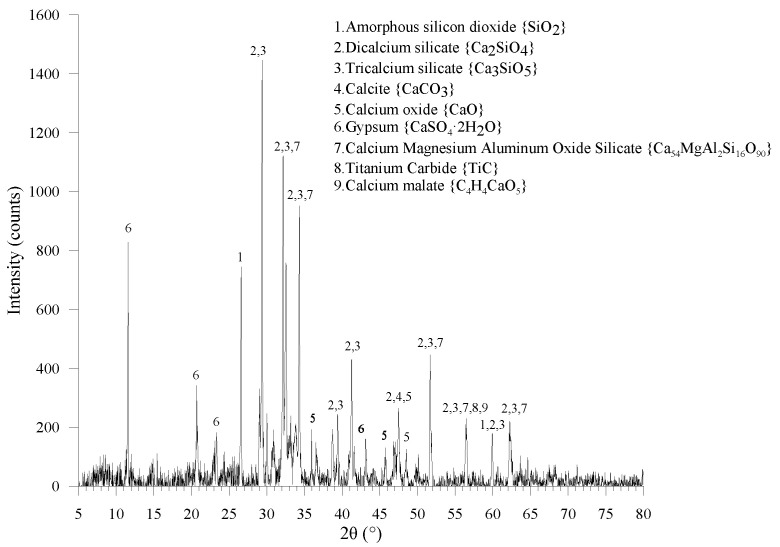
X-ray Diffraction results of cement used.

The chemical composition of cement used (P.O. 42.5 as per Chinese Standard, Common Portland Cement, in GB 175 [31]) is shown in Table 1 and the XRD curve in Figure 2.

Sharp characteristic peaks in XRD curves indicate that the main mineral compositions are amorphous silicon dioxide (SiO_2_), dicalcium silicate (C_2_S), tricalcium silicate (C_3_S), calcite (CaCO_3_), calcium oxide (CaO), and gypsum (CaSO_4_∙2H_2_O). It is also noticed that calcium magnesium aluminum oxide silicate (Ca_54_MgAl_2_Si_16_O_90_), titanium carbide (TiC), and calcium malate (C_4_H_4_CaO_5_) exist in the cement clinker. River sand with a 1.69 fineness modulus and 2.0 mm maximum diameter was utilized. The major chemical, physical, and mechanical properties of PVA fiber used are listed in Table 2.

**Figure 3:**
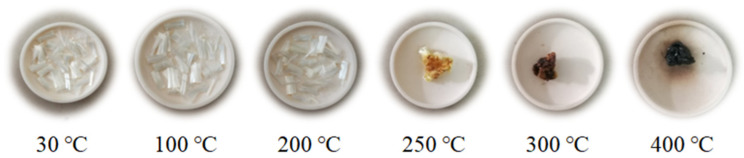
Morphologies of polyvinyl alcohol fiber under elevated temperatures.

PVA fibers belong to the type of thermoplastic material produced by the melt spinning method [32]. It is commonly believed that the thermal condition is detrimental to the PVA fiber morphology, especially when the temperature exceeds its melting point [33]. The morphologies of PVA fibers under temperatures from ambient conditions to 400 °C are displayed in Figure 3.

**Figure 4:**
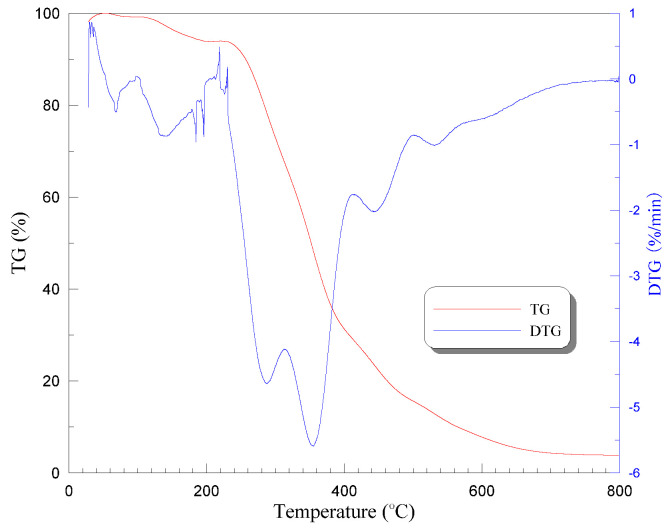
Thermogravimetric and derivative thermogravimetry curves of PVA fiber used.

It is clear that no significant change occurred up to 200 °C, but when the temperature was elevated to 250 °C and above, an evident color change of PVA fiber was found together with the follow-up of thermal decomposition, remaining a foam-like residue at 400 °C finally. Herein, the PVA fiber exhibited a yellow appearance at 250 °C, and brown and black at 300 and 400 °C, respectively. The heat sensitivity of PVA fibers can further be illustrated by derivative thermogravimetry (DTG). Figure 4 shows the thermogravimetric (TG) and DTG curves of PVA fiber, indicating a melting point of approximately 235 °C and thermal instability between 220 °C and 420 °C.

The mixture proportion and strength properties of ECC containing the above PVA fibers are shown in Table 3.

As can be seen, the water-to-cement ratio (W/C) was 0.45 and the sand-to-cement ratio (S/C) was 0.80. PVA fibers were incorporated in a volume fraction of 2.0%. The flexural strength of such PVE-ECC was evaluated according to ISO 679 [34] as 7.2 ± 1.4 and 8.9 ± 0.4 MPa at 7 and 28 days, respectively, while the compressive strength was 27.6 ± 2.1 and 35.4 ± 1.5 MPa at 7 and 28 days, respectively.

### 2.2. Specimen Preparation and Curing

Six groups in total of 18 cubic specimens of 100 × 100 × 100 mm^3^ were designed and cast to investigate the behavior of PVA-ECC under ambient temperature, 200, 400, 600, 800, and 1200 °C. Specifically, the test items included surface response, weight loss, compressive strength, composition deterioration behavior, and microstructure change of PVA-ECC. All specimens were prepared in one batch to ensure uniformity. The mixing procedure was consistent with ISO 679 [34] and previous research, cf. [35,36]. After casting, matrixes were wrapped with polythene sheets at room temperature for 24 h before demolding. After that, all specimens were moved to a standard curing chamber with a constant temperature of 20 ± 2 °C and relative humidity over 95%.

### 2.3. Heating Regimes

**Figure 5:**
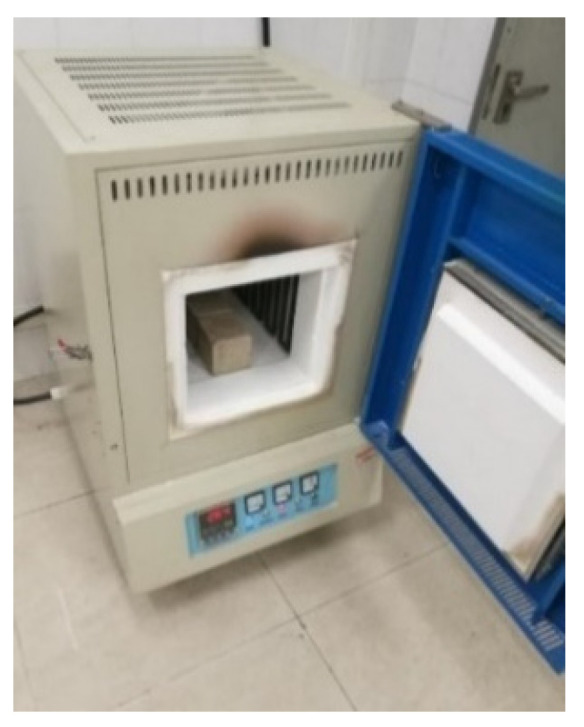
Muffle furnace with the function of programming control.

After 56 days’ curing, the specimens were taken out from the curing chamber for testing. Before heating, they were wiped with dry towels to a state of saturated surface. Then, the cubes were divided into 5 groups and heated in a muffle furnace with the function of programmed control (see Figure 5) to target temperatures.

**Figure 6:**
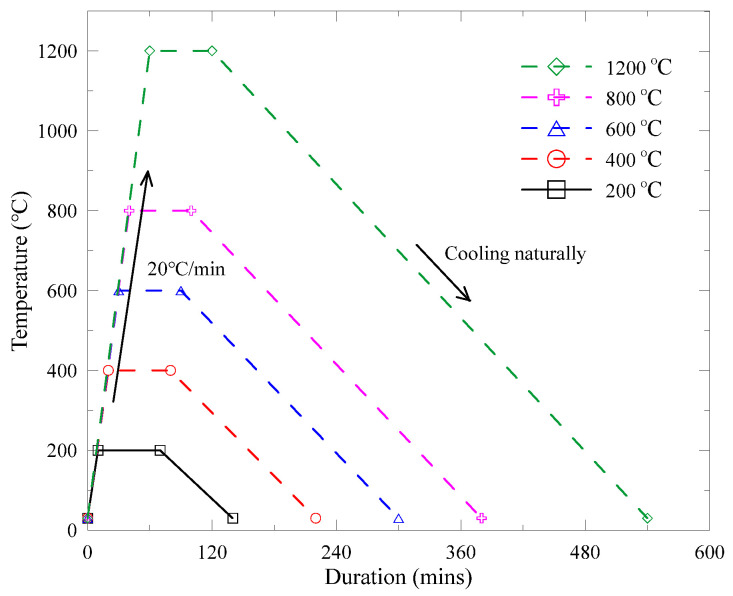
Heating regimes of various target temperatures.

The heating regimes are plotted in Figure 6 as temperature–duration curves, achieving 200, 400, 600, 800, and 1200 °C with a heating rate of 20 °C/min.

The target temperature was kept for 1 h to guarantee a relative thermal stable state for both the furnace and tested specimens. After that, the specimens were cooled down in air to ambient temperature.

### 2.4. Test Methods

TGA 4000 thermogravimetry with a temperature accuracy of ±0.1 °C and sensitivity of 0.1 μg was applied to analyze the weight change of the specimen with the increase in temperature. A high-magnification electron microscope was used to observe the surface cracking behavior after heating. The maximum magnifying power was 800×. Furthermore, after residual mechanical properties test, the samples were made into slides and powders with a hammer and triturator. Slides were used to analyze the morphology and microstructure with a JEOL-JSM-7001F (Tokyo, Japan) scanning electronic microscope. Meanwhile, the powders were used for XRD analysis with an X-ray diffractometer (40 kV and 40 mA Cu X-ray tube, Almelo, Netherlands) with *2θ* from 5° to 80° scope in a 0.03° step-length.

## 3. Results

### 3.1. Color and Appearance Changes

**Figure 7:**
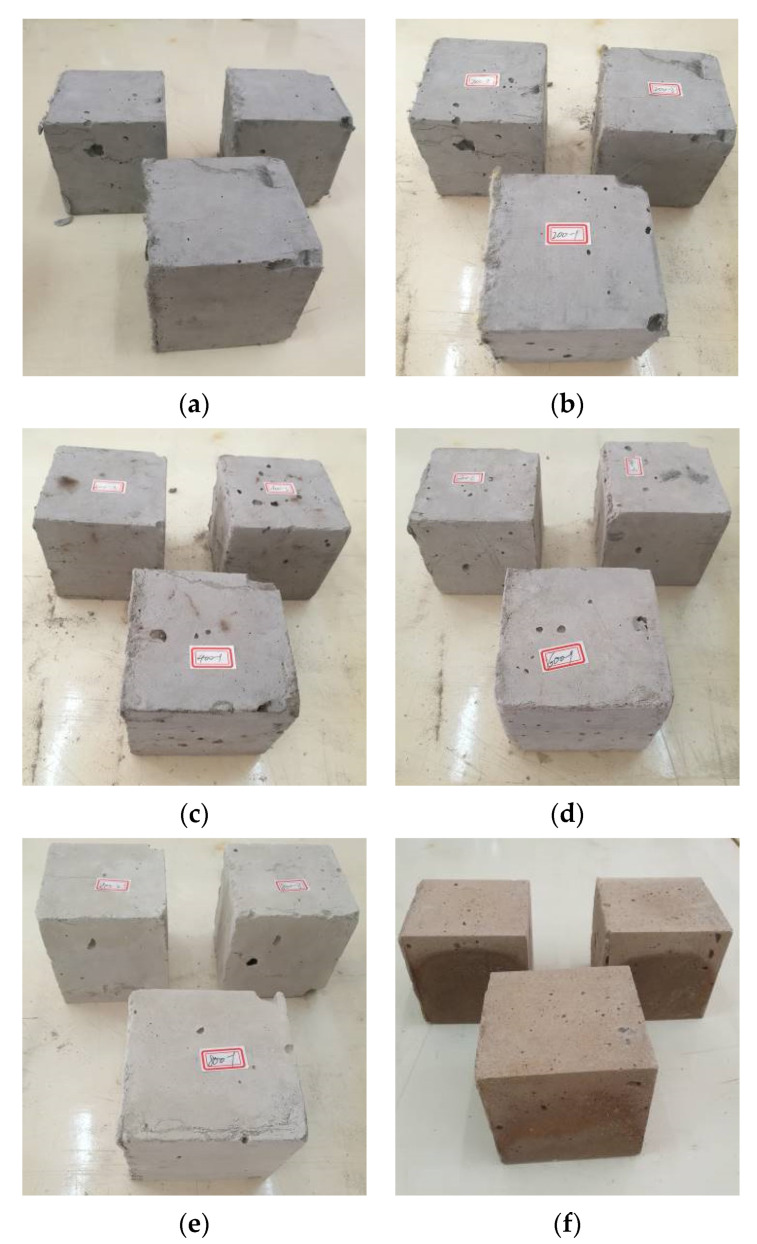
Appearance of cubic specimens after exposure to various temperatures: (**a**) 30 °C, (**b**) 200 °C, (**c**) 400 °C, (**d**) 600 °C, (**e**) 800 °C, (**f**) 1200 °C.

Visual observations of cubic specimens after one-hour exposure to various temperatures are displayed in Figure 7.

Apparent color changes were found with increasing temperature. Herein, the surfaces of samples appeared grey at room temperature, light-grey at 200 °C, and whitish-grey at 400 °C. However, as the temperature increased to above 600 °C, the color turned to yellowish-grey. Similar color variances were also observed in [25,28]. Such transitions in appearance are possibly related to the physicochemical changes of composites, including water evaporation, chemical decomposition of hydration products, and fiber melting. For instance, the color of PVA fibers changed from transparent white at room temperature to yellow at 250 °C and dark black at 400 °C, which might affect the appearance of cubic specimens. Huang et al. [37] reported *β*-dicalcium silicate and mullite at around 400 to 600 °C for the ultra-lightweight cementitious composite, and hence appear deep red instead. At 800 °C, the cubes appeared light brown. Interestingly, the color turned brownish at 1200 °C (Figure 7f), mainly attributed to the redox reaction of iron oxides or hydrous iron oxides. 

### 3.2. Surface Cracking and Spalling Behavior

**Figure 8:**
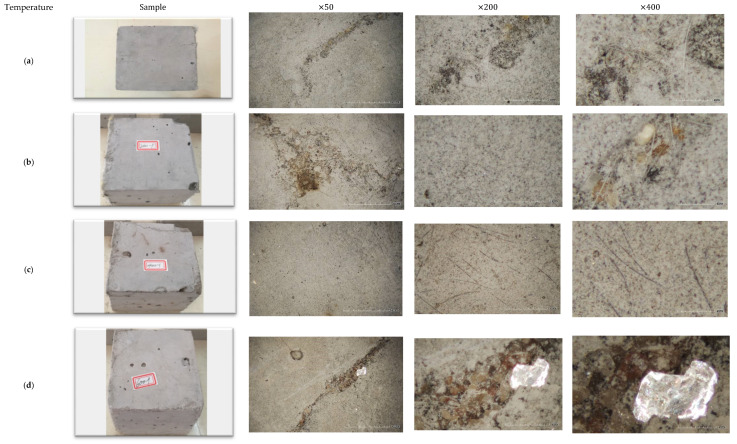

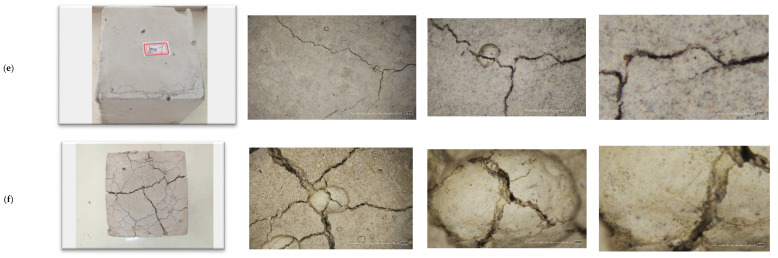
Surface characteristics of cubic specimens after exposure to various temperatures: (**a**) 30 °C, (**b**) 200 °C, (**c**) 400 °C, (**d**) 600 °C, (**e**) 800 °C, (**f**) 1200 °C.

Thermal stresses generate gradually due to water evaporation, thermal expansion, and drying shrinkage when cementitious materials are under fire/high temperatures. Once stress surpasses the tensile resistance of cementitious composites, surface cracking occurs and propagates as the heating temperature continues to increase. Figure 8 shows the surface crack patterns of cubic PVA-ECC specimens.

First, no surface cracking was found at temperatures below 400 °C, while hairline cracks were observed by the naked eye at 600 °C (see Figure 8d, ×50). Other researchers [25,28,38,39] also observed apparent hairline cracks when the heating temperature exceeded 400 °C. Herein, hairline cracks appeared around hydration products first and then developed along unhydrated cement grains. Meanwhile, it is noticeable that a new crystalline substance was formed at this level of high temperature. As the temperature was elevated to 800 °C, the microcracks propagated rapidly, resulting in longer crack lengths and coarser crack widths, cf. [27,40,41,42]. The surface cracking became more severe at 1200 °C, and reticular cracks and upheavals appeared clearly, as shown in Figure 8f. Two mechanisms may be responsible for this; one is the additional shrinkage deformation (evidenced by the length change as per [41]), and the other is the decomposition of hydration products. Such a chemical reaction might alter the pore structure inside the composite due to the filling effect. Stresses induced by shrinkage and pore pressure synergistically cause stress redistribution in the specimens; once magnified over capacity, the surface area inevitably cracks [29].

Spalling, generally exhibited as a sudden ejection of fragment, is a catastrophic failure mode when cementitious composites encounter fire/high temperatures. Concrete with high strength is prone to explosive spalling [43] due to two mechanisms [44]: Restrained thermal dilation and increased pore pressure. As the PVA fibers used in the present study melt at approximately 235 °C, explosive spalling behavior was effectively prevented. Similar conclusions were drawn in previous literatures [45,46,47]. Herein, the pressure relief mechanism of polymeric fiber in cementitious composites contributes to the nonspalling behavior even at high temperatures of 1200 °C. Specifically, pore pressure can be relieved due to the reservoir effect of air bubbles or micro-cracks around fibers. Thus, the network channels formed beyond the fiber melting point, consequently accommodating the expanding steam and moisture-vapor migration [44,48,49]. Interestingly, when the micro fibers melted at around 235 °C, an irritating odor was smelt from the heating muffle in the meantime.

### 3.3. Weight Loss

**Figure 9:**
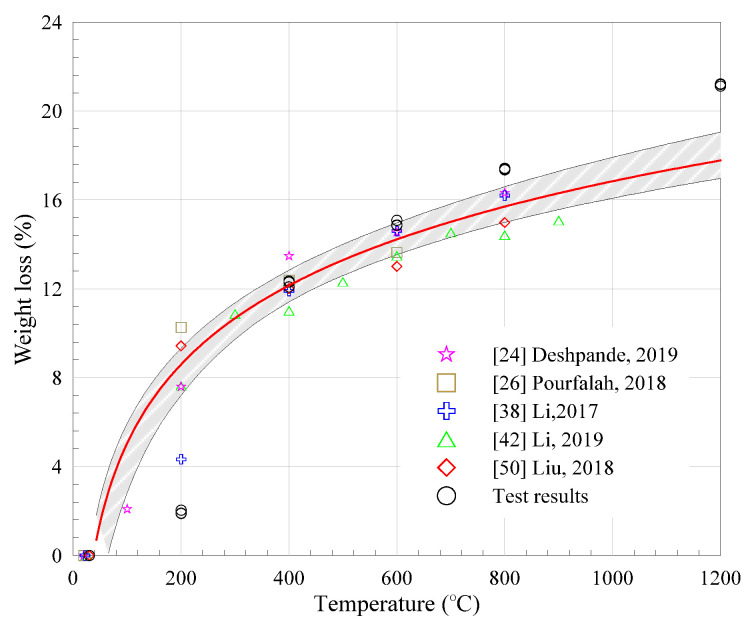
Weight loss at different temperatures from the literature and present study.

The loss in weight is one of the noticeable responses for ECC specimens under fire conditions and has been investigated by many researchers at various elevated temperatures. Figure 9 summarizes the mass loss of PVA-ECC with various compositions from literatures and test results [24,26,38,42,50].

**Figure 10:**
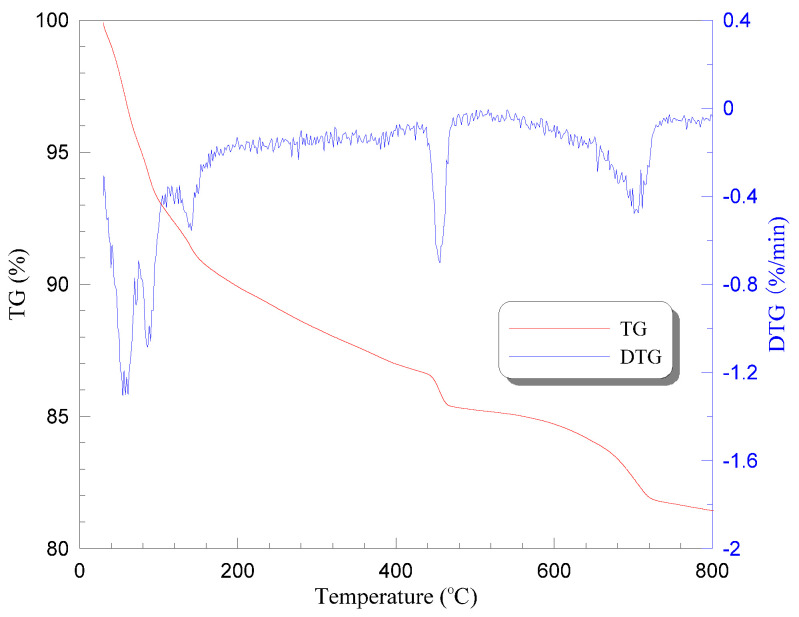
TG and DTG curves of PVA-ECC tested.

A similar trend is observed that weight loss increased with the heating temperature, whereas the increasing gradient decreased in the meantime. The average weight loss ratio of tested specimens (three in a group) was evaluated as 1.94%, 12.25%, 14.94%, 17.39%, and 21.18% after exposure to temperatures of 200 °C, 400 °C, 600 °C, 800 °C, and 1200 °C, respectively. TG and DTG curves of PVA-ECC also confirmed such weight change, as shown in Figure 10.

Herein, evaporation of water may be the main reason for the weight loss below 200 °C. As the temperatures increase up to 400 °C, the loss of chemically bound water and hydrates decomposition (indicated by sharp drop in DTG curves) may further contribute to the mass change of ECC specimens together with the thermal pyrolysis of PVA fibers [38]. Thereafter, extensive microcracks started to generate in the specimens when the heating temperature increased to above 600 °C and, consequently, led to higher weight loss, cf. [25]. Particularly, more than 17% weight was lost when the surrounding temperature reached 800 °C, mainly attributed to the decomposition reactions [37,48], discussed in Section 4.1.

Based on the existing data, the prediction model of mass loss rate (*β_m_*) with heating temperature (*T*) is provided in Equation (1) for PVA-ECC. A good agreement could be found between the test results and the prediction model. In the absence of actual data, Equation (1) might be applied to empirically predict the remaining weight of PVA-ECC after exposure to elevated temperature.

(1) $$\beta_{m}=5.122\times \ln\left(T\right)-18.538,\;R^{2}=0.85$$

### 3.4. Residual Compressive Strength

**Figure 11:**
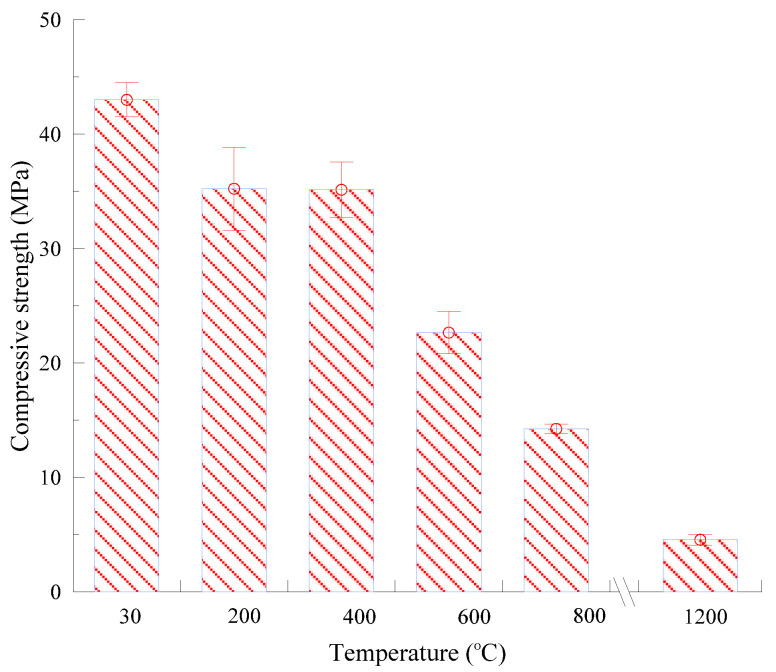
Compressive strength of PVA-ECC specimens at ambient and high temperatures.

Strength loss seems to be one of the main reasons for the collapse of concrete structures under fire. Figure 11 shows the compressive strength of PVA-ECC under ambient and high temperatures.

Compared to the control group, all specimens subjected to elevated temperatures exhibited lower strength under compression loads. Specifically, the average residual compressive strength of three specimens was evaluated as 35.2, 35.1, 22.7, and 14.2 MPa after exposure to 200, 400, 600, and 800 °C, respectively. A dramatic decrease of bearing capacity was found at 600 °C, which was also detected by Peng and Huang [30]. When the preheated temperature increased to 1200 °C, the strength dropped to 4.6 MPa, indicating that PVA-ECC completely lost its resistance to external load. A total decreasing tendency was observed in strength with temperature, which is somehow different from the phenomena obtained in published papers. A strength enhancement effect at the temperature range of 200 to 400 °C was generally observed by other researchers [28,39,51,52]. Such discrepancy may be caused by a higher W/C and the absence of fly ash in the present study. Herein, an appropriate high temperature was found to accelerate the hydration of unhydrated cement clinkers and fly ash particles [53], thus producing more C-S-H gels. Hence, the pore structures and compactness or density of ECC at such temperatures were improved [25,29,54]. In the present study, higher W/C left a smaller amount of unhydrated cement clinkers, especially when the curing age increased to 56 days for the tested specimens. Therefore, the compressive strength did not show an increase in comparison with that of 43.0 MPa at room temperature.

**Figure 12:**
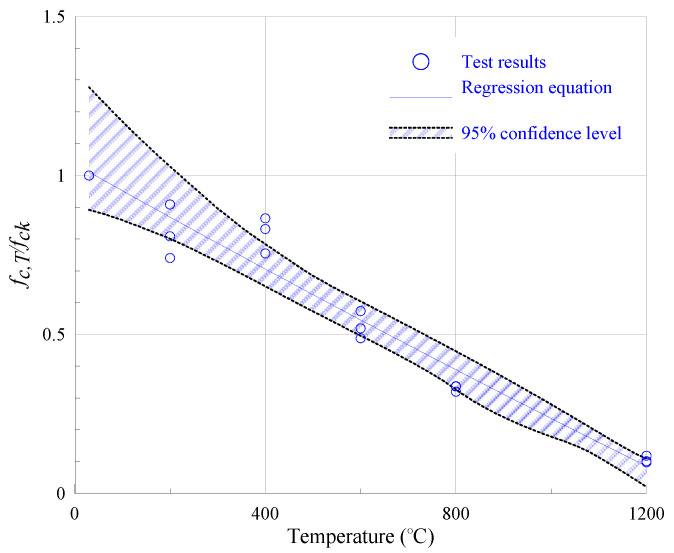
Relative residual strength at various temperatures.

Generally, the ratio of strength at elevated temperatures (*f_c,T_*) to strength at room temperature (*f_ck_*) is defined as the relative compressive strength (*f_c,T_/f_ck_*) to estimate the deterioration rate of post-exposure specimens. The average values of *f_c,T_/f_ck_* in the present study are 81.9%, 81.7%, 52.7%, 33.1%, and 10.6% at 200 °C, 400 °C, 600 °C, 800 °C, and 1200 °C, respectively (see Figure 12).

Compared to normal concrete at a similar strength level in [24], PVA-ECC exhibits more promising thermal resistance up to 800 °C despite the melting of fiber around 235 °C. Moreover, a regression equation is provided as Equation (2) to predict the residual strength of PVA-ECC.

(2) $$\frac{f_{c,T}}{f_{ck}}=1.0362-0.0008T+4.242\times 10^{-8}T^{2}\;\left(30\;{}^{\circ}C\leq T\leq 1200\;{}^{\circ}C\right)$$

The shadow area in Figure 11 represents the confidence interval of the regression equation at the 95% confidence level. As can be seen, the regression equation agrees well with the experimental data, and the determination coefficient is as high as 0.94.

### 3.5. Failure Modes

**Figure 13:**
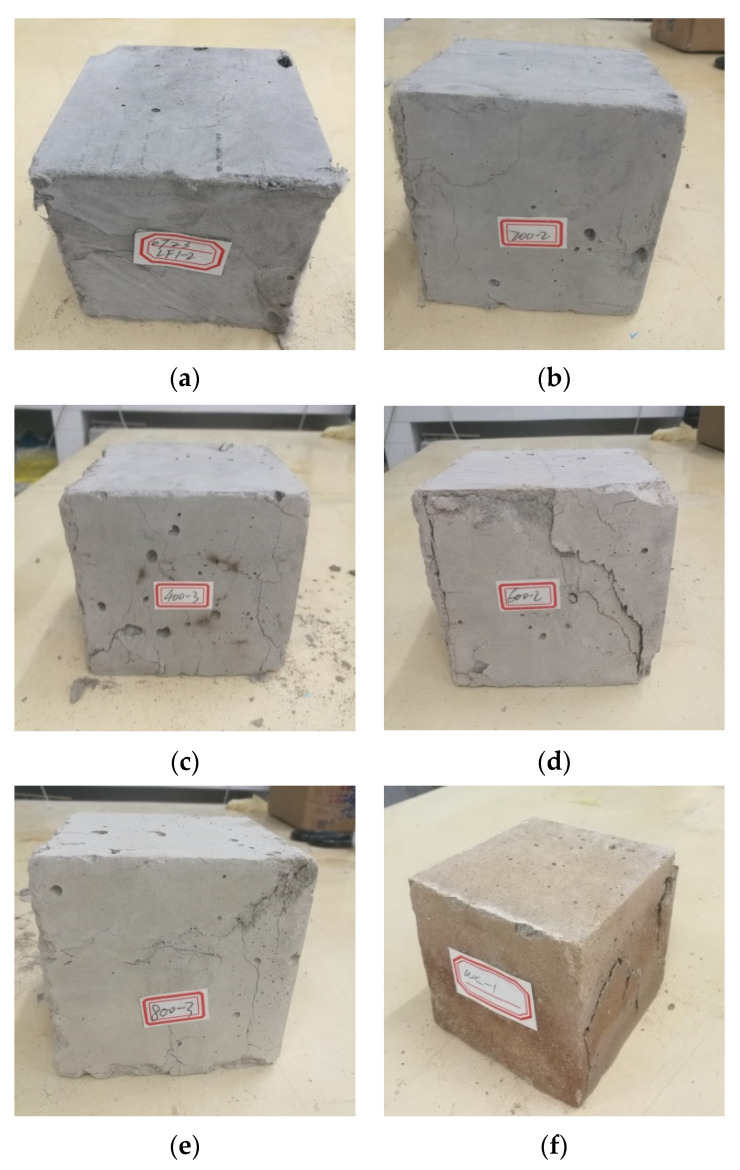
Failure modes of post-exposure PVA-ECC cubes after compression tests: (**a**) 30 °C, (**b**) 200 °C, (**c**) 400 °C, (**d**) 600 °C, (**e**) 800 °C, (**f**) 1200 °C.

After compression tests, the failure modes of fire-damaged cubic specimens are displayed in Figure 13.

It is clear that no obvious cracks were observed in the surfaces of damaged cubes at room temperature (see Figure 13a). At 200 °C, only multiple microcracks were found. As the temperature increased, failure behavior became more severe and brittle, exhibiting larger and longer cracks together with black burn spots on the surface of 400 °C -heated cubes, shown in Figure 13c. When the temperature increased to 600 °C, surface flaking was observed under compression load and exhibited a brittle nature, forming a cone residue just like normal concrete. The main reasons for failure behavior deterioration were considered to be the moisture evaporation and initial crack propagation. Such effects worsened along with the increase in heating temperature [37,38]. Particularly for cubes exposed to 1200 °C, transverse cracks on the surface were formed, and thus, led to evident crushing behavior after reaching maximum compression load (see Figure 13f).

## 4. Microstructure Analysis

### 4.1. Chemical Compositions

**Figure 14:**
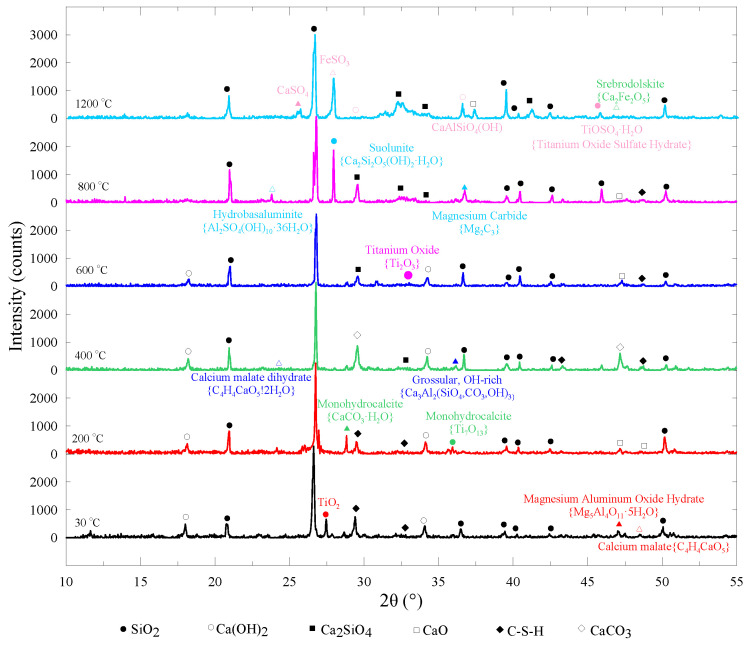
XRD results of PVA-ECC after exposure to various temperatures.

The most significant hydration products in cement matrix are commonly believed as C-S-H and CH. The phase compositions of PVA-ECC can be clearly examined by XRD instruments. Tested curves are shown in Figure 14.

As can been seen, C-S-H and CH are clearly observed at the sharp characteristic peaks on the tested intensity–degree curve at ambient temperature, cf. [37]. As the samples contain plenty of silica sand, silicon dioxide (SiO_2_) can be detected at most of the peaks despite the heating temperatures. For CH, no evident change in XRD patterns for 30, 200, 400, and 600 °C was found, but when the heating temperature was elevated up to 800 °C and above, calcium hydroxide was not detected any more. This indicates that the decomposition of CH initiated in the temperature range of 400 °C to 600 °C and finished beyond 600 °C, cf. [25,30]. By contrast, C-S-H was found at an initial decomposition at 200 °C and completely decomposed at 800 °C within 1 h of heating, which explained well the severe deterioration of residual strength in PVA-ECC at such temperature levels [27,30]. Furthermore, calcium carbonate (CaCO_3_) appeared evidently at 400 °C as a reaction product of C-S-H decomposition under elevated temperature, monohydrocalcite (CaCO_3_∙H_2_O) (see XRD curve of 200 °C) decomposition, and calcium oxide (CaO) carbonation. However, CaCO_3_ was no longer observed at temperatures above 600 °C. Instead, CaO was found beyond 600 °C, which implies that decomposition of CaCO_3_ initiated at around 500–550 °C under higher pressure conditions in a heated cementitious system. In addition, dicalcium silicate (Ca_2_SiO_4_) had been generated when the heating temperature increased to 400 °C and above, the amount of which increased as the temperature further elevated. Hence, the above chemical changes at high temperatures undoubtedly altered cracking behavior and pore structure, consequently leading to the deterioration in macro mechanical strength, especially when the temperature exceeded 800 °C, cf. [50].

It was also noticed that titanium dioxide (TiO_2_), calcium malate (C_4_H_4_CaO_5_), and magnesium aluminum oxide hydrate (Mg_5_Al_4_O_11_∙5H_2_O) existed in the tested samples. Herein, TiO_2_ turned into monohydrocalcite (Ti_7_O_13_) at 200 °C, titanium oxide (Ti_2_O_3_) at 600 °C, and titanium oxide sulfate hydrate (TiOSO_4_∙H_2_O) at 1200 °C, which indicate deoxygenation reaction at high temperature as the ratio of O to Ti decreased from 2:1 at 30 °C to 13:7, 3:2, and 1:1 at 200, 600, and 1200 °C, respectively. C_4_H_4_CaO_5_ turned into calcium malate dehydrate (C_4_H_4_CaO_5_∙2H_2_O) at 400 °C as a result of moisture evaporation and high temperature, but could be observed beyond 600 °C. As for Mg_5_Al_4_O_11_∙5H_2_O, it decomposed after a heating of 200 °C, and a more stable reaction product of magnesium carbide (Mg_2_C_3_) was found at 800 °C instead. Such variations in compositions of ECC inevitably led to a weaker interfacial transition zone and coarser pore structures. Grossular with rich –OH (Ca_3_Al_2_(SiO_4_,CO_3_,OH)_3_) appeared at 400 °C, and suolunite (Ca_2_Si_2_O_5_(OH)_2_∙H_2_O) and hydrobasaluminite (Al_2_SO_4_(OH)_10_∙36H_2_O) at 800 °C. When the heating temperature was elevated to 1200 °C, calcium sulphate (CaSO_4_), ferrous sulfite (FeSO_3_), and srebrodolskite (Ca_2_Fe_2_O_5_) were detected in the XRD curve. Interestingly, brown vuagnatite (CaAlSiO_4_(OH)) was also found at 1200 °C, which may explain well the apparent color change mentioned in Section 3.1.

### 4.2. Micro-Morphologies

**Figure 15:**
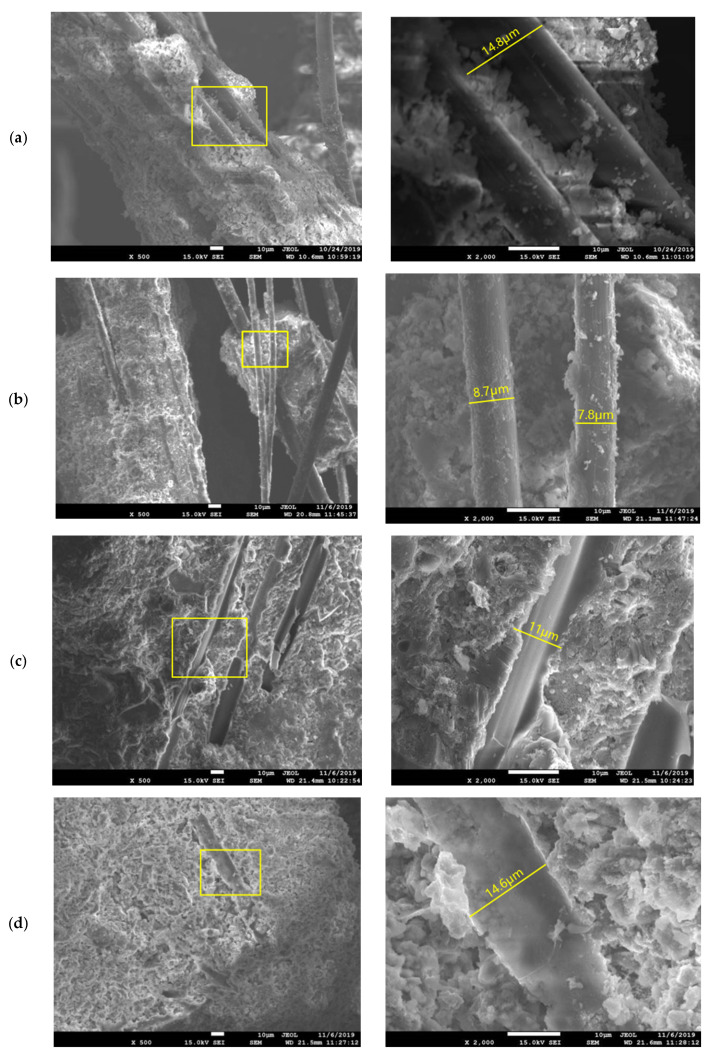

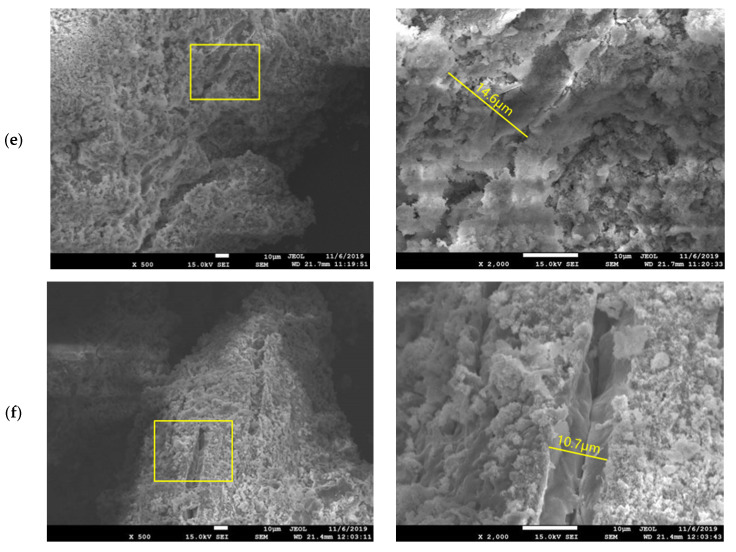
Morphologies of PVA-ECC after exposure to various temperatures: (**a**) 30 °C, (**b**) 200 °C, (**c**) 400 °C, (**d**) 600 °C, (**e**) 800 °C, (**f**) 1200 °C.

The behavior deterioration of PVA-ECC is believed to be in close connection with the structure change under the microscope. Figure 15 shows the morphologies of post-exposure cubic specimens under SEM observations.

It is clear that at room temperature, PVA fibers bond tightly with the matrixes, resulting in an enhanced interfacial transition zone compared to normal concrete [55]. Such an adhesion effect is attributed to the hydrophilic hydroxyl groups in the PVA chain. When the temperature increased to 200 °C, no evident detriment was found and the fibers remained intact with no rupture on the surfaces. However, a reduction in the diameter of fibers was observed mainly due to the burning behavior of PVA, measured as 8.7/7.8 μm (see Figure 15b) in comparison with that of 14.8 μm (Figure 15a) at 30 °C. Such a shrinking in size might inevitably detach the fiber and matrixes around, thus weakening the interfacial transition zone and consequently lowering the mechanical properties of PVA-ECC in macro scale.

After exposure to 400 °C, no fiber was ever observed in the micrographs of PVA-ECC. Instead, longilineal needle-like channels were left after the melting procedure of PVA fibers (see Figure 15c). These channels provided additional passages for water evaporation and, hence, prevented the explosive spalling of cementitious composites [37,42,56]. The channels exhibited almost the same size with the distributed fiber, whereas the diameter in Figure 15c was measured as 11 μm because the wrapped cement paste covered the channel. The longitudinal groves engraved in the surface imply that the pullout process was accompanied by severe plastic deformations, which might improve the ductility of the composites simultaneously. Such a change in fracture surface also indicates that the fiber-matrix bond strength decreased with temperature and might not be strong enough to transfer higher tensile stress as fibers at room temperature do [57]. Meanwhile, decomposition of C-S-H was initiated at this level of temperature, forming crystalline solid and network structures near the channels, and enlarging the pore volume in the meantime, cf. [51].

When the heating temperature increased to 600 °C, the dents left by fibers became smooth while the corresponding width almost remained the same (14.6 μm in Figure 15d). In terms of hydration products decomposition, the degree became significantly higher, exhibiting irregular bush-like structures under the microscope [52]. Herein, the average pore diameter and total pore volume were increased as large particles formed around the nucleus, demonstrated as a pore coarsening effect in [25,27,52]. Furthermore, at 800 °C, the channels were gradually filled with newly produced substance. The size of dents was measured as 14.6 μm, which became harder to find in the micrographs, shown in Figure 15e. Meanwhile, evident microcracks were observed in the heated cement matrixes due to the loss of their crystal structure, indicating a prominent decrease in mechanical properties of cementitious composites. Finally, at 1200 °C, a more homogenous distribution of reaction products was displayed in Figure 15f. It is clear that channels left by PVA fibers were narrowed and the average width decreased to 10.7 μm. Three mechanisms might be responsible: Thermal expansion effect, volume expansion effect, and pore coarsening effect. Herein, the thermal expansion effect refers to the expansion of the substance under high temperatures, which enlarges with the increase in surrounding temperature. The volume expansion effect is caused by the gain in products volume after expansive chemical reactions due to the chemical composition variation under high temperatures. The third probable reason could be the pore structure coarsening effect, generated by the evaporation of water/moisture or release of gases inside the matrixes.

A similar microstructure change has also been found by other authors [28,58] when studying the thermal response of traditional ECC containing a high volume of fly ash. They observed that numerous fly ash particles exhibited noncrystallized or amorphous structures at 600 °C, whereas the particles completely melted at 800 °C, forming micro pores and wrapping the dehydrated C-S-H gels closely, cf. [37]. SEM analysis of the present study showed no such phenomenon, and also, the microcracking behavior of matrix under high temperature was found mitigated because no fly ash particles were provided for crack propagation along the weak interfaces between the spherical cenosphere and cement paste.

## 5. Conclusions

This paper concentrates on the behavior deterioration of polyvinyl alcohol fiber-reinforced cementitious composite (PVA-ECC) after exposure to high temperatures. The following conclusions can be drawn accordingly:

- The color appeared grey at room temperature, light-grey at 200 °C, whitish-grey at 400 °C, yellowish-grey at 600 °C, light-brown at 800 °C, and brownish at 1200 °C, respectively.
- Hairline cracks were observed beyond 600 °C and surface cracking became more severe with the increase in heating temperature.
- Explosive spalling behavior was effectively prevented in the presence of PVA fibers due to the channels left after the melting of fibers around 235 °C.
- The average weight loss ratio of tested specimens was evaluated as 1.94%, 12.25%, 14.94%, 17.39%, and 21.18% after exposure to temperatures of 200 °C, 400 °C, 600 °C, 800 °C, and 1200 °C, respectively.
- The average values of *f_c,T_/f_ck_* were 81.9%, 81.7%, 52.7%, 33.1%, and 10.6% at, 200, 400, 600, 800, and 1200 °C, showing a monotonic decreasing tendency.
- Calcium hydroxide decomposed initially in the range of 400–600 °C and finished beyond 600 °C, while C-S-H began at around 400 °C and completely decomposed at approximately 800 °C.
- A reduction in diameter of fibers was observed at 200 °C while longilineal needle-like channels were found left beyond 400 °C. Dents were gradually filled with the newly produced substance due to the synergistic effects of thermal expansion, volume expansion of chemical reactions, and pore structure coarsening.

## Figures and Tables

**Table 1:** Chemical compositions of cement used.

Composition	CaO	SiO_2_	Al_2_O_3_	SO_3_	Fe_2_O_3_	MgO	K_2_O	TiO_2_	MnO	Na_2_O	P_2_O_5_	SrO	LOI
Cement	50.30	23.18	6.88	2.49	2.48	2.14	0.68	0.42	0.15	0.10	0.09	0.03	11.03

**Table 2:** Properties of PVA fiber used.

Fiber	Chemical Formula	Chemical Structure	Density (kg/m^3^)	Length (mm)	Diameter (mm)	Strength (MPa)	Modulus (GPa)	Elongation at Break (%)	Melting Point (°C)
PVA	(C_2_H_4_O)_n_	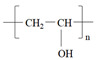	1300	12	0.015	1780	39	7.0	235

**Table 3:** Mixture properties of PVA-engineered cementitious composite (ECC) studied (1 m^3^).

Item	PVA-ECC
Cement (C) (kg/m^3^)	926
Sand (S) (kg/m^3^)	741
Water (W) (kg/m^3^)	417
PVA fiber (kg/m^3^)	26
W/C	0.45
S/C	0.80
7-day flexural strength (MPa)	7.2 (1.4)
7-day compressive strength (MPa)	27.6 (2.1)
28-day flexural strength (MPa)	8.9 (0.4)
28-day compressive strength (MPa)	35.4 (1.5)

Note: Numbers in brackets are standard deviations in MPa.
